# Serpentine Soils Do Not Limit Mycorrhizal Fungal Diversity

**DOI:** 10.1371/journal.pone.0011757

**Published:** 2010-07-23

**Authors:** Sara Branco, Richard H. Ree

**Affiliations:** 1 Committee on Evolutionary Biology, University of Chicago, Chicago, Illinois, United States of America; 2 Field Museum of Natural History, Chicago, Illinois, United States of America; 3 Centro de Investigação de Montanha, Bragança, Portugal; University of California Riverside, United States of America

## Abstract

**Background:**

Physiologically stressful environments tend to host depauperate and specialized biological communities. Serpentine soils exemplify this phenomenon by imposing well-known constraints on plants; however, their effect on other organisms is still poorly understood.

**Methodology/Principal Findings:**

We used a combination of field and molecular approaches to test the hypothesis that serpentine fungal communities are species-poor and specialized. We conducted surveys of ectomycorrhizal fungal diversity from adjacent serpentine and non-serpentine sites, described fungal communities using nrDNA Internal Transcribed Spacer (ITS) fragment and sequence analyses, and compared their phylogenetic community structure. Although we detected low fungal overlap across the two habitats, we found serpentine soils to support rich fungal communities that include representatives from all major fungal lineages. We failed to detect the phylogenetic signature of endemic clades that would result from specialization and adaptive radiation within this habitat.

**Conclusions/Significance:**

Our results indicate that serpentine soils do not constitute an extreme environment for ectomycorrhizal fungi, and raise important questions about the role of symbioses in edaphic tolerance and the maintenance of biodiversity.

## Introduction

In terrestrial ecosystems, soil conditions are important determinants of biodiversity both above and belowground, influencing the ecology and evolution of plants [1], fungi [2], animals [3], and other organisms [4]. Serpentine soils, derived from ultramafic rocks, exhibit low levels of essential macronutrients, low calcium-magnesium ratios, and toxic concentrations of chromium, nickel, and other elements [5], [6]. These soils offer inhospitable abiotic conditions that limit biological establishment and diversity.

The vast majority of research on the ecology and evolution of serpentine species has focused on plants, yielding abundant evidence of colonization constraints. In general, serpentine plant communities have low levels of productivity, species richness, and plant cover. They tend also to be differentiated in vegetation type from neighboring areas and have high rates of endemism [5]–[10]. In addition, adaptive divergence in plant species found across serpentine and non-serpentine soils has been demonstrated via reciprocal transplants [11]. Serpentine soils are also a strong selective agent for other organisms, and serpentine adaptive radiations have been found both in snails [12] and caddisflies [13].

While it is well known that the vast majority of plants tolerant of serpentine soils are involved in mycorrhizal associations [7], [14], very little is known about serpentine communities of mycorrhizal fungi. Serpentine characteristics are most likely detrimental for fungal establishment as high levels of heavy metals are almost universally toxic [15], affecting fungi directly through enzymatic inhibition and disruption of cellular integrity [16], and indirectly through the production of free radicals [17]. Our study is focused on ectomycorrhizal (ECM) fungi. These are symbiotic with vascular plants, live in the soil and associate with their photosynthetic partner at the root level, mediating root uptake of water and nutrients and receiving carbohydrates in return [14]. ECM fungi have been hypothesized to play a major role in facilitating the acclimation of plants to local soil conditions, including tolerance to high heavy metal concentrations [18]. Specifically, serpentine-adapted ecotypes of ECM fungi have been suggested and hypothesized to be critical to the success of trees in colonizing serpentine soils [19]. Despite such suggestions of fungal serpentine specialization, it is still unclear how serpentine soils affect ECM fungal diversity. Preliminary field surveys showed no detectable particularities on serpentine ECM fungal communities [20], [21], however, such studies were based on limited sampling and are insufficient for drawing conclusions on the existence of fungal serpentine adaptation.

We surveyed ECM fungal communities from adjacent serpentine and non-serpentine oak forests in northeastern Portugal. In an attempt to fully characterize the fungal communities, we focused on a simple system, with a single host tree species and a small geographic range, and used an intensive sampling regime, with a sampling effort an order of magnitude higher than previous studies. We developed a novel methodology based on the nrDNA Internal Transcribed Spacer (ITS) region to compile community phylogenetic trees and analyze the phylogenetic community structure of ECM fungi.

**Figure 1:**
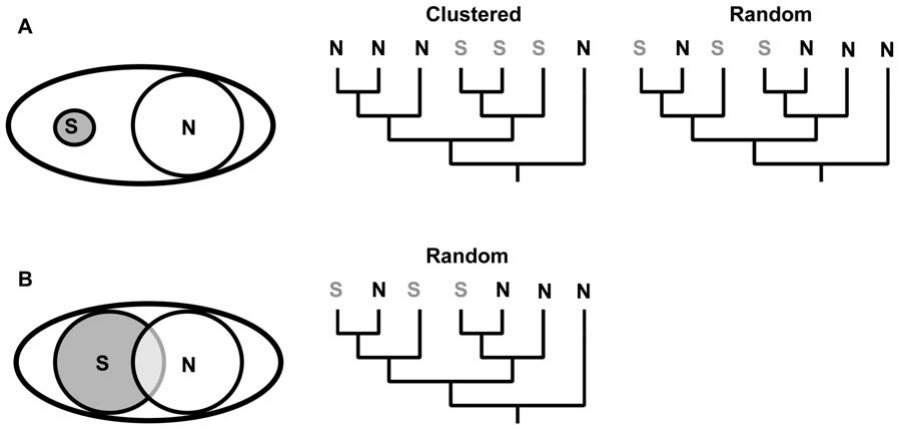
Hypothesized scenarios for serpentine and non-serpentine ECM fungal communities. Serpentine (gray circle) and non-serpentine (white circle) alpha diversity (with circle size as a measure of higher species) in relation to the regional species pool (white oval), the change in species composition across soil type (beta diversity) and the phylogenetic relationships among serpentine (S) and non-serpentine (N) taxa. A–Low serpentine alpha diversity, high beta and phylobetadiversities across soil types, indicating serpentine soil is an environmental filter. The serpentine community can be phylogenetically clustered or random, depending if traits underlying specialization are conserved or not. B–Similar alpha diversity and low beta and phylobetadiversities across soil types, suggesting serpentine and non-serpentine communities are a random sample of the regional species pool and serpentine soil does not constitute an environmental filter.

We hypothesized that serpentine soils are a strong selective agent for ECM fungi and impose barriers to colonization. To test this hypothesis, we compared alpha diversity, beta diversity, and phylogenetic beta diversity (“phylobetadiversity” [22]) of natural ECM fungal communities on serpentine and non-serpentine soil. Phylobetadiversity quantifies how phylogenetic relationships among species change across communities and allows an evolutionary approach in evaluating community structure [22]. We expected to find low alpha diversity associated with serpentine soil, as well as high beta and phylobetadiversity across soil types (Fig 1A). We predicted finding serpentine taxa occurring as sister-groups to non-serpentine taxa, a pattern consistent with speciation events associated with local adaptation to serpentine environments. To the extent that functional traits conferring serpentine tolerance are phylogenetically conserved, we predicted phylogenetic clustering, i.e., serpentine species more closely related than expected by chance [23], consistent with the idea that serpentine soil is difficult to colonize, but once colonized promotes adaptive radiation. If functional traits are not conserved, we would expect more random phylogenetic structure. The null hypothesis is that serpentine soils do not constitute a stressful environment for ECM fungi. In this case, we expect serpentine and non-serpentine communities to be indistinguishable from random draws from the regional species pool, with equivalent alpha diversity, and low beta diversity and phylobetadiversity (fig 1B).

Serpentine ECM fungal communities were found to be as diverse as non-serpentine and composed by the same fungal lineages. Our results do not support serpentine soil as an environmental constraint for ECM fungi.

## Results

Soil analyses confirmed the serpentine character of the serpentine sites: low calcium to magnesium ratio, high magnesium content, and high levels of nickel and cromium (table 1). All tested parameters were significantly different across serpentine and non-serpentine soil, except lead and zinc.

**Table 1:** Average soil chemical composition of serpentine and non-serpentine (with standard deviations; ppm –parts per million; % BS - Percent Base Saturation, * - significant p value of the one-way ANOVA analysis).

Soil parameter	Serpentine soil	Non-serpentine soil
Al (ppm)	12.3 (±5.4)	28.8 (±0.7)
B (ppm)*	1.1 (±0.1)	0.3 (±0.0)
C (%)*	10.7 (±1.6)	1.6 (±0.2)
Ca (%BS)	15.9 (±2.0)	42.9 (±3.3)
Ca (ppm)*	1014.3 (±89.5)	1349.8 (±83.4)
Ca/Mg*	0.4 (±0.0)	2.0 (±0.0)
Cation Exchange Capacity*	32.3 (±2.1)	16.6 (±0.9)
Cd (ppm)*	0.38 (±0.1)	0 (±0.0)
Cr (ppm)*	0.5 (±0.1)	0.2 (±0.1)
Cu (ppm)	0.1 (±0.1)	0.4 (±0.1)
Fe (ppm)*	23.3 (±4.8)	6.9 (±0.8)
K (%BS)*	0.7 (±0.2)	2.6 (±0.40
K (ppm)*	81.3 (±21.7)	154.5 (±26.2)
Mg (%BS)*	60.4 (±5.7)	22.4 (±8.6)
Mg (ppm)*	2378.8 (±327.6)	430.5 (±171.8)
Mn (ppm)	180.6 (±44.0)	117.8 (±7.1)
N (%)	0.5 (±0.1)	0.2 (±0.0)
Ni (ppm)*	29.8 (±8.1)	1.0 (±0.4)
NO_3_-N (ppm)	7.8 (±3.3)	1.3 (±0.0)
P (ppm)*	27.3 (±3.9)	7.5 (±2.1)
Pb (ppm)	31.7 (±0.3)	30.5 (±0.0)
pH*	6.1 (±0.1)	5.3 (±0.1)
Zn (ppm)	1.9 (±0.9)	1.1 (±0.5)

**Figure 2:**
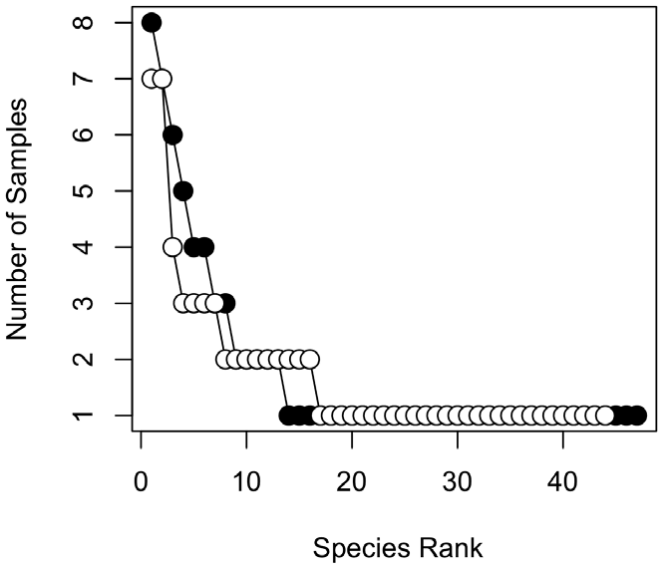
Sequence rank/frequency curves. Serpentine (closed circles) and non-serpentine (open circles) ECM fungal communities based on sequence data (species defined at a 95% sequence similarity cut-off).

**Figure 3:**
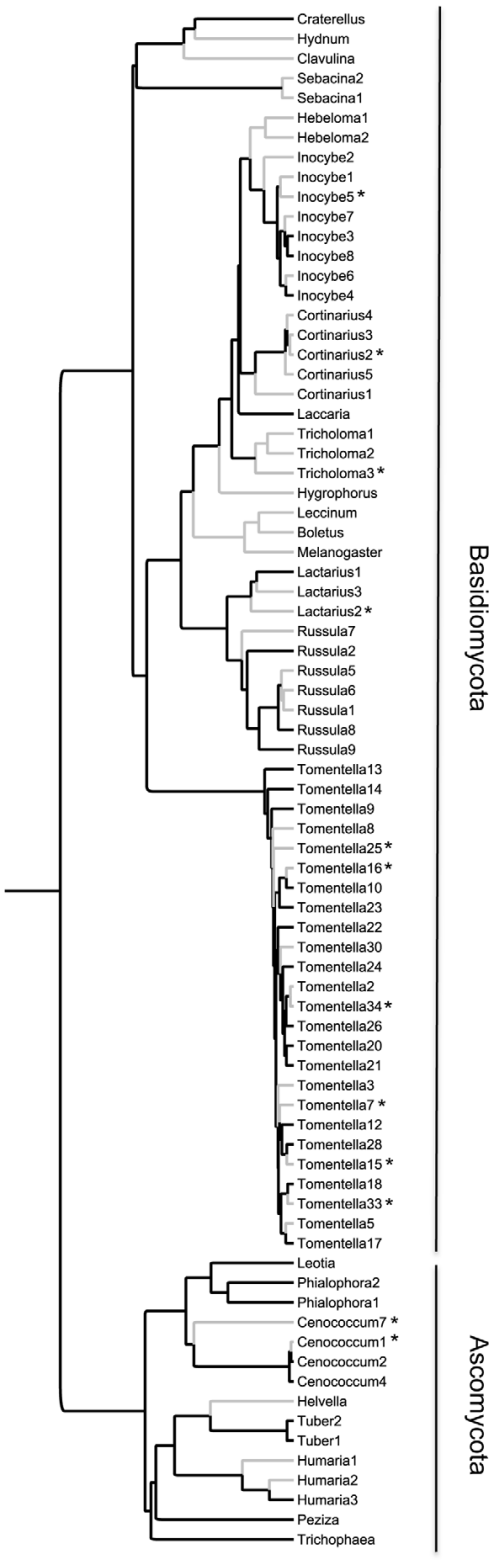
Fungal community phylogenetic tree. Hierarchical phylogenetic supertree based on nrDNA LSU and ITS sequence data showing the phylogenetic relationships of the ECM fungal species detected in serpentine (grey branches) and non-serpentine soil (black branches) (* indicates species detected in both soil types).

The serpentine and non-serpentine forests revealed very diverse ECM fungal communities, with similar fungal richness and community structure, yet little fungal overlap. We found forty-three species belonging to fourteen genera on the non-serpentine forests and forty-five species from sixteen genera on the serpentine habitat (figs. 2 and 3). Only 15% of all species were shared between the two habitats. In both communities, fungi tended to be rare, with the majority found only in a single sample (fig. 2). Few fungi were also shared between the two serpentine and the two non-serpentine forests (15% in the former and 16% in the latter). There was a similar number of genera exclusive to one soil type, and it is interesting that Boletales (*Boletus, Leccinum*, and *Melanogaster*) were detected only on serpentine forests (fig. 3).

**Figure 4:**
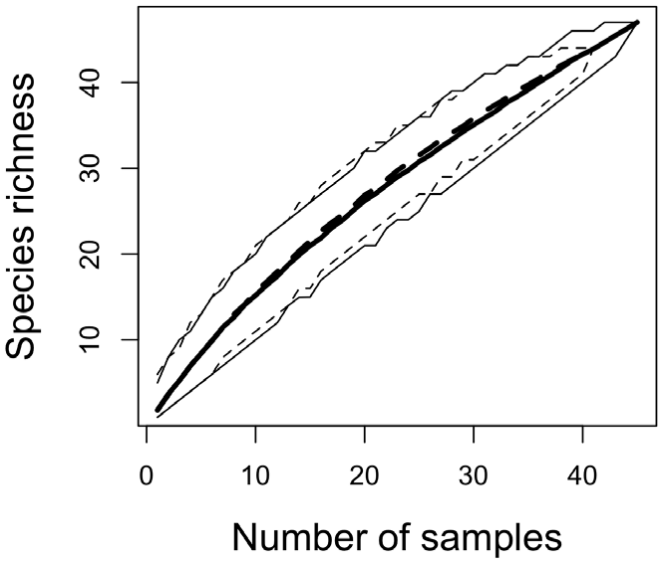
ECM fungal species accumulation curves. Species accumulation curves based on sequence data with 95% bootstrap confidence intervals for the serpentine (black line) and non-serpentine (dashed line) communities (species defined at a 95% sequence similarity cut-off).

**Figure 5:**
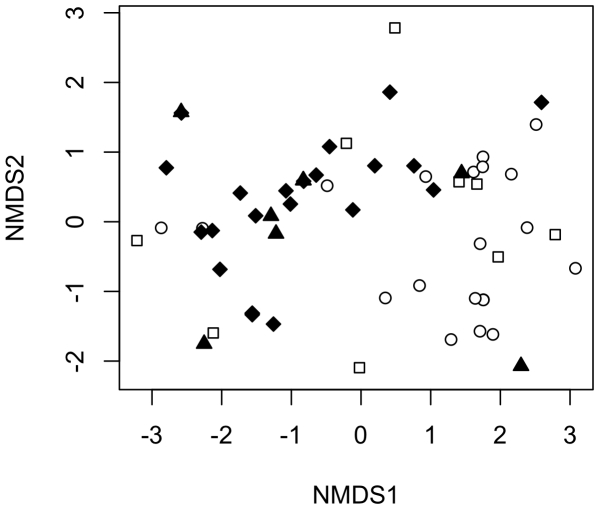
ECM fungal community ordination plot. Non-metric dimensional scaling plot for species frequencies in each forest based on the sequence data. White circles–non-serpentine Rabal; white squares–non-serpentine Petisqueira; black diamonds–serpentine Serra da Nogueira; black triangles–serpentine Espinhosela. Stress = 14.04.

The serpentine and non-serpentine species accumulation curves were indistinguishable, with no signs of saturation and overlapping confidence intervals (fig. 4). Although the communities were clearly different, the ordination analysis did not group the samples by soil type or forests (fig. 5).This indicates that although there were very few species shared across serpentine and non-serpentine sites, the fungal communities are not statistically distinguishable.

Fungi from serpentine and non-serpentine forests were phylogenetically diverse, with representatives of all major ECM fungal lineages such as Agaricales, Boletales, Russulales, Thelephorales, and several genera within the Ascomycota (fig. 4). Analyses on the community phylogenetic structure showed no support for phylogenetic clustering of fungal species in both communities (serpentine: NRI = 1.28, *p* = 0.12, NTI = 1.62, *p* = 0.06; non-serpentine: NRI = 0.20, *p* = 0.6, NTI = −0.28, *p* = 0.68).

## Discussion

Our study indicates that serpentine soils impose no detectable constraints on the richness or phylogenetic diversity of ECM fungi. These results are unexpected, given prior knowledge about serpentine soils as an extreme environment and their effect as a selective agent on several groups of organisms.

ECM fungi are hyperdiverse and assemble in complex and dynamic communities that are difficult to characterize [24], [25]. Highly diverse ECM fungal communities dominated by rare taxa have been documented from a variety of habitats and geographic locations [25]–[27], and communities associated with oak trees seem to be particularly rich [28]–[31]. Although we studied a very simple forest system, with a restricted geographical range and a single host tree species, we still found fungal communities to be very diverse. Extreme environments tend to host low diversity [32], and low ECM fungal diversity has been hypothesized under harsh environmental conditions [24].

However, we did not find a depauperate serpentine community. This study is based on extensive sampling and multiple methodological approaches and although we were not able to fully describe the ECM fungal communities and found many fungi in single samples, we report equally complex serpentine and non-serpentine communities. There are very few studies on serpentine ECM fungi, however analyses based on more limited sampling had previously indicated ECM fungal communities from serpentine sites followed similar patterns as communities from non-serpentine environments [20], [21]. Here, more than just corroborating this finding, we use a phylogenetic framework to report the lack of evidence for serpentine soils as a strong evolutionary agent for ECM fungi. A similar fungal rich serpentine pattern has been described for arbuscular mycorrhizal fungi [33], [34], suggesting this trend of high serpentine richness is widespread across the fungal kingdom.

The wide phylogenetic distribution of species within ECM fungal communities indicates that either ECM fungi are pre-adapted to inhabit serpentine soils, suggesting these habitats are not cause for evolutionary change, or that the evolution of serpentine tolerance has been extraordinarily labile, arising independently in a diverse array of fungal lineages. The lack of evidence for radiations of ECM fungal serpentine specialists contrasts with other extreme environments in which specialized clades have evolved, such as fungi in tundra soils [2], Antarctic fish [35], desert lizards [36], plants in several extreme environments [37], and snails and caddisflies in serpentine soils [12], [13]. It is difficult to draw conclusions about the functional traits underlying serpentine tolerance. Phylogenetic clustering is evidence of habitat filtering when functional traits are conserved [38], however phylogenetic structure alone is not sufficient to make inferences both on ecological process and trait pattern [39]. Because we did not find phylogenetic clustering on serpentine soil we can rule out the possibility of habitat filtering associated with trait conservatism. More information on which functional traits are relevant as well as their evolutionary patterns is needed for a thorough understanding of the effect of serpentine soil on ECM fungi.

Serpentine soils pose three major challenges to potential colonists: low Ca/Mg ratio, high heavy metal content and nutrient deficiency [5], [7], [8]. These factors might be mitigated by the biology of ECM fungi. It has been described that fungi show high constitutively tolerance to heavy metals [40], and *in vitro* studies have unraveled several different mechanisms involved with tolerance, revealing the existence of a range of fungal physiological strategies for coping with heavy metals [41], [42]. ECM fungi can, therefore, be invulnerable to the chemical particularities of serpentine environment. On the other hand, the studied serpentine soil contains high levels of nitrogen and phosphorus, compared to the non-serpentine soil. This higher fertility might contribute to the lack of fungal serpentine specialization.

ECM fungi are symbiotic and receive their energy from their photosynthetic partners [14]. Although serpentine soils may offer a challenging environment, plant partners still provide the necessary amount of carbohydrates to support rich and diverse serpentine ECM fungal communities. In fact, plant hosts have been suggested to act as buffers for ECM fungi in extreme environments, providing the necessary nutrition for fungal survival and preventing harsh conditions from strongly affecting them by [27].

Preliminary evidence suggests that environmental constraints imposed by serpentine soils are significant for the plant host, *Quercus ilex* subsp. *ballota*. Seedlings of serpentine origin grow significantly less compared to non-serpentine seedlings [43], and although there is still no conclusive evidence for serpentine local adaptation in this oak species, there are definitely marked physiological differences between seedlings from serpentine and non-serpentine origin. Despite these differences, oaks might be allocating similar amounts of carbon to their fungal partners, contributing to a generalized fungal serpentine establishment. It is very interesting that growth differences in the plant host are not reflected in the richness and phylogenetic diversity of their ECM fungal communities.

Serpentine soils do not constitute a major selective agent for ECM fungi and do not lead to higher-level differentiation between serpentine and non-serpentine fungi. Alternatively, as suggested by [19], there might be specialization at or below the species level. The fact that most ECM fungi found were rare does not give insight on this matter – although there was little fungal serpentine/non-serpentine overlap, the majority of species were detected only once, making it difficult to assess if they are specific to a soil type. ECM fungal serpentine local adaptation with edaphically specialized populations was not addressed in this study. However, results from a reciprocal transplant experiment where serpentine and non-serpentine fungi from the same study sites sampled in this study were subject to native and non-native soil, showed no evidence of serpentine soil as a physiological barrier for the establishment of ECM fungi, suggesting absence of serpentine specialization (Branco, unpublished).

Our results raise important questions about how symbiotic relationships shape the diversity of extreme environments. Specialization to serpentine soils might be occurring at the level of plant ecotypes, meaning that it is the plant that facilitates ECM fungal colonization of serpentine soils. Conversely, constitutive or adaptive tolerance to heavy metals by ECM fungi might be the key factor mediating plant serpentine colonization. The physiological mechanisms of serpentine tolerance in fungi remain obscure and are in need of further research. Teasing apart these relationships and mechanisms will not only yield insight of evolutionary interest, but will also lead to more effective strategies for restoration biology and bioremediation.

## Materials and Methods

### Study sites

Ectomycorrhizal (ECM) fungal communities were sampled from two serpentine and two non-serpentine *Quercus ilex* subsp. *ballota* (Desf) Samp. forests located in Trás-os-Montes, northeastern Portugal. *Quercus ilex*, a Mediterranean evergreen oak, is the only tree species tolerant to serpentine soils in the region [44]. The serpentine oak forests are located at Serra da Nogueira (41°47′58.975 N, 6°54′15.545 W) and Espinhosela (N 41°51′22.126 W 06°50′424), while the non-serpentine forests are located in Rabal (N 41°52′14.875, W 06°44′40.000) and Petisqueira (N 41°51′.53.635 W 06°31′43.945). This region has been exposed to anthropogenic disturbance for many centuries and the studied forests have been used by the local populations as a source of wood and as a place to feed cattle (mostly sheep). The non-serpentine forests grow on chromic luvisols [45].

Sampled sites were not more than 20 km apart. All the forests are monospecific, composed of mature oak trees. *Cistus ladanifer* L. is the only other ECM plant host present and showed a low density in all sampled forests. Soil analyses were conducted for the Serra da Nogueira and Rabal sites in 2005. We analyzed four soil samples per forest, each consisting of the combination of 5 sub-samples collected 5 m apart. Standard soil parameters, macro- and micronutrients, and heavy metal contents were analyzed (pH, N, C, Al, P, K, Ca, Mg, B, Mn, Zn, Cu, Fe, Pb, Ni, Cr, Cd, NO_3_-N, cation exchange capacity, percent base saturation for K, Mg, and Ca). Analyses were conducted at the University of Massachusetts Soil and Plant Tissue Testing Laboratory (Amherst, USA), except for C and N that were performed at Argonne National Laboratory and pH, which was measured in the Soil Laboratory of Escola Superior Agrária de Bragança (Portugal). Soil nitrogen and carbon analysis were performed using a LECO CN-2000 analyzer (LECO Corporation, St. Joseph, MI, USA); all remaining elements were analyzed using a modified Morgan extraction and ICP (Spectro Analytical Instruments, Fitchburg, MA, USA). Serpentine and non-serpentine soils were compared using a standard one-way ANOVA.

### ECM community sampling

We defined two 50 m perpendicular transects in each forest and selected a focal tree every 5 m, totaling twenty focal trees per forest. We collected a 1000 cm^3^ (10×10×10 cm) soil cube in the vicinity of each focal tree. Serra da Nogueira and Rabal were sampled in two consecutive years (2005 and 2006 for the first and 2006 and 2007 for the latter) and Petisqueira and Espinhosela were sampled once in 2008. All samples were collected in the spring, between May and June, when oak leaves develop. We retrieved ECM oak root tips from refrigerated soil cubes within four days after collection and sorted samples under a dissecting microscope into morphotypes (based on mantle and external hyphae morphology). One root tip from each morphotype present in each soil cube was dried using silica gel and saved for molecular analyses. Many morphotypes occurred recurrently throughout the different soil samples and were therefore analyzed molecularly more than once. We analyzed an average of four root tips per soil sample (ranging from three to six) and a total of five hundred and thirty three root tips. We obtained fungal samples from 62 soil cubes.

### Molecular protocols

All collected root tips in the four sampled forests were sequenced (593 root tips). Amplifications obtained from the samples collected between 2005-2007 were sequenced using ITS1F and ITS4 primers [26], [46] following [28] protocol and screened using an ABI 3730 DNA analyzer. Samples collected in 2008 were extracted and amplified as before, except without fluorescent-tagged ITS primers and sequenced as above. The sequencing success was in the order of 65%; we grouped the readable clean sequences using a conservative 95% similarity cut-off [47], [48]. Each set of sequences was considered a different fungal species and the consensus sequence was saved and compared with the NCBI database using the BLAST algorithm. All sequences matched fungal genera, however only the ones confidently assigned to a genus (with at least the first 20 BLAST hits belonging to one genus) were retained for analyses (GenBank accession numbers FJ897173 to FJ89250; table S1). Species frequency (soil cube presence/absence) matrices were generated for each habitat and used to assess ECM fungal richness and frequency.

### Community analysis

To investigate differences in the fungal communities associated with each soil type, we conducted multidimensional scaling analyses with the sequence data. Due to the low fungal frequencies both data sets were disconnected (many samples did not share any fungal species) and 22 samples in the sequence data were discarded. Analyses were implemented in the vegan package of R [49], [50].

### Community phylogenies of ECM fungi

The species of ECM fungi identified from root tips belonged to a wide range of taxa within Ascomycota and Basidiomycota. In order to estimate the phylogenetic diversity of communities sampled within and between sites, we constructed a species-level phylogenetic tree using a hierarchical approach involving three steps described below: 1) compilation of a genus-level tree, 2) compilation of a species-level tree for each of the included genera, and 3) grafting the species-level trees onto the genus-level tree.

#### Genus-level tree

We defined the phylogenetic relationships between the detected genera based on recent published studies on the phylogenetic systematics of fungi [51]–[60]. To estimate branch lengths on this topology, we assembled a molecular data set for divergence-time analysis. Sequences of nuclear ribosomal large subunit (LSU) DNA were retrieved from GenBank for each genus found in our survey, and for an outgroup, *Glomus intraradices* (table S2). Genera were assumed to be monophyletic, except for *Russula* and *Lactarius* [56], which were grouped together. Since *Cenococcum* is a complex genus that encompasses much genetic diversity and is suspected to include several distinct lineages [61], LSU sequences for each species of *Cenococcum* in our sample were generated using the LR0R, LR6 and LR3 primers [62] and included in a Dothidiomycete phylogeny (G. Mugambi and S. Hundhorf, unpublished). These species formed a monophyletic group (data not shown) and one sequence (FJ897251) was selected and used in the genus-level tree. One *Russula* LSU sequence was included in the genus-level tree. Branch lengths for the genus-level topology were first estimated from the LSU dataset using PAUP* [63] by maximum likelihood, under the HKY85 model of nucleotide evolution. Next, they were adjusted by nonparametric rate smoothing [64] using the software package APE [65].

#### Species-level trees

For each genus, a data set of nuclear ribosomal ITS sequences was assembled that included outgroup sequences from its sister group in the genus-level tree. Data sets were aligned using ClustalX [66]. For each data set, a maximum likelihood tree was computed using Garli [67] with the default settings. Table S1 shows which sequences were used in each phylogeny including ITS sequences of congeneric species from GenBank. The species-level trees were rooted in Mesquite [68] and made ultrametric as above.

#### Assembling the final fungal community tree

For each species-level tree branch lengths were scaled to be common with the LSU genus-level tree and outgroups and GenBank sequences were pruned. To account for differences in molecular rates of evolution between ITS and LSU, genus-specific scaling factors were estimated by comparing branch lengths separating ingroup species from a common outgroup. Species-level trees were then grafted on the genus-level tree (scaling and grafting script available from the authors upon request).

### Community phylogenetic structure

The phylogenetic structure of the serpentine and non-serpentine ECM fungal communities was assessed using the Net Relatedness Index (NRI) and the Nearest Taxon Index (NTI) [23]. NRI and NTI compare the observed phylogenetic distances for the taxa included in the communities under study with a distribution generated by the distances between species sampled at random from the tree. These indices indicate if species in a community are phylogenetically more closely related (clustered; >0) or less closely related (overdispersed, <0) than expected by chance. When NRI or NTI equal zero, species in a community are random regarding their phylogenetic relationships. NRI and NTI were calculated using Phylocom [69], with the comstruct function, randomization method 3 and 999 runs.

## Supporting Information

Table S1 ITS sequences used to compile each of the species-level fungal phylogenies.
(0.04 MB XLS)

Table S2 nrDNA Large Subunit sequences used to compile the genus-level fungal phylogeny. * - Outgroup
(0.03 MB XLS)
